# Polymers-Based Biosensors and Bioelectronics: Designs and Applications

**DOI:** 10.3390/bios16040211

**Published:** 2026-04-09

**Authors:** Vinh Van Tran

**Affiliations:** Department of Mechanical Engineering, Gachon University, Seongnam 13120, Republic of Korea; vanvinhkhmtk30@gmail.com or bom19881205@gachon.ac.kr

## 1. Introduction

Biosensors and bioelectronics (B&B) are typical devices working at the interfaces of biology and electronics. The design and fabrication of biosensors that can provide a real-time manner to monitoring electrophysiological signals as well as metabolite of biofluids such as sweat, tear, saliva, and interstitial fluid have been considered as one of the most promising strategies for development of bioelectronic devices and human health monitoring systems [1]. Among diverse kinds of B&B devices, wearable biosensors with strong flexibility and stretchability have been recently attracted tremendous interest as next generations in diagnosing the physiological and biochemical activities of tissues and organs, as well as various medical conditions [2]. Moreover, wearable biosensors possess a high possibility in providing continuous and real-time manner for dynamic and noninvasive measurements of biochemical markers in biofluids [3]. Wearable sensors integrated into smartphones and other mobile devices, referred to as smart wearable biosensors, can also provide valuable insights for the precise control and monitoring of human health at the point of care (POC). Over the past decade, smart wearable biosensors have been envisioned as one of the most important biomedical devices for the early and accurate monitoring of human diseases and the protection of human health. Many studies have attempted to design and fabricate next-generation B&B devices, driving a new revolution in wearable bioelectronics [4,5]. These innovative B&B devices have demonstrated some outstanding characteristics and superior advantages, including simultaneous monitoring of multiple factors in both human health and the environment, good biodegradability and biocompatibility, high reliability, and miniaturization. Among numerous advanced B&B devices, electronic skin (e-skin) and implantable biosensors have recently explored as patient-friendly diagnostic technologies. Depending on the specific applications and the target body fluids, B&B devices could be constructed in a wide range of wearable platforms [6], including: (i) Epidermal wearable biosensors (e.g., gloves, patches, and skin-worn sensors) are designed to comfortably conform to human skin for detecting target biomolecules in human sweat and interstitial fluid; (ii) Microneedle devices with minimal invasiveness can continuously monitor interstitial fluid biomarkers; (iii) Wearable electrochemical biosensors integrated into smartwatches or wristband have been recently designed and used for detecting metabolites, nutrients, and glucose levels in sweat and interstitial fluid; (iv) Eye wearable biosensors such as contact lenses and eyeglasses are designed for sensing specific biomarkers in tears.

Recently, integrated B&B devices, constructed with three main components—a biosensor, a power source, and a signal transmission unit—have been more extensively developed and implemented in practical applications. However, the power sources in these devices are regarded as major obstacles, significantly limiting their development and applicability, as they reduce mechanical flexibility and increase the size of the devices [7]. Especially, implantable B&B devices often require extra power source for long-term operation, thereby greatly limiting service life of devices and reducing user’s comfort. Moreover, the replacement of batteries even requires multiple surgeries, offering a big challenge for their practical applications. Therefore, development of the power supply has been considered as one of the most important strategies for significantly extending the practical applications of B&B devices

Polymers have shown some superior advantages over other materials such as metals, metal oxides, carbon-based materials, and ceramic materials for B&B applications [8,9]. They possess mild synthetic conditions, large scalable processing, diverse structures, high flexibility, low working temperature, and biodegradation and biocompatibility. The progress and innovations in nanotechnology have offered new opportunities for the design and fabrication of different novel polymer structures in biosensing applications. For further expanding applications of polymers in the fabrication of B&B devices, several key requirements need to be adapted, including: (i) the sensing performances such as sensitivity, response/recovery time, and reversibility/reproducibility of response must be significantly improved by varying the chemical structures and size of the polymers [10]; (ii) the selectivity toward specific biomarkers is crucial for precise detection. The development of polymer nanoparticles with increased large and effective surface area has been regarded as the most important and promising approach for enhancing the response rate as well as sensitivity [11]. Over the past decades, advancements in nanotechnology have significantly contributed to the rapid development of polymeric nanoparticles for use in biosensors. The chemical or physical properties of polymers have been significantly improved and optimized in various nanoparticle forms and morphologies, offering the highest sensing performances. Moreover, polymeric nanoparticles could be engineered with a wide range of surface functional groups as well as bioconjugates for improving the biocompatibility [12]. Recently, polymers have been extensively employed to synthesize stimuli-responsive polymeric hydrogels as a potential platform for flexible and stretchable B&B devices in human health monitoring [13]. Due to their high water content, the stimuli-responsive hydrogels exhibit excellent biocompatibility and biodegradability, combined with elasticity, durability, and transparency, making them suitable for the fabrication of wearable or implantable B&B devices [14].

Among various polymer materials, conjugated polymers (CP), high molecular weight compounds with conjugated structure, are the most common polymers used for the design and fabrication of B&B devices [15]. In the past two decades, CPs have witnessed tremendous advances and innovations in providing optimal structures with the highest performance for biosensing applications. In comparison with other common materials such as metal/metal oxides and carbon-based materials, CPs show better solution processability and high mechanical flexibility, offering them as ideal materials for large scale production and fabrication of B&B devices such as transistors [16]. In addition, CPs also exist the instinct characteristics of polymers, i.e., softy, stretchability, and mechanical comfortability, make them proper candidates for wearable and stretchable bioelectronic devices. Organic field-effect transistors (OFETs) and organic electrochemical transistors (OECTs) are the most common electronic devices fabricated from CP materials for biosensor applications. Among them, OECTs are often preferred for construction of biosensors due to their operation in aqueous environment and superior performances in the amplification of low signals [17]. Moreover, the OECTs provide an appealing and effective approach for the design of highly sensitive and flexible B&B devices. The sensing mechanism of a typical OECT biosensor mainly relied on the reduction/oxidation reactions of the analytes on the gate electrode [18]. Therefore, the sensing performance of OECTs is independent of specific instruments or spectrometers, allowing them to be designed and fabricated as miniaturized devices. Furthermore, the transistor-based biosensors possess intrinsic amplification capability, high magnifying minor signals from trade amount of biomarkers, thereby effectively enhancing the limits of detection (LOD) [19]. The OECTs constructed from CP semiconductors exhibit high biocompatibility, low operating voltages, and high disposability on flexible substrates, providing significant advantages for OECT-based biosensors. In summary, the development and innovation of CP materials in OECT-based B&B devices represent promising strategies for current and future research.

Electronic tongues (e-tongues) and electronic skin (e-skin) has appeared as the latest advances in the development of biosensor and bioelectronics technologies [20,21]. E-tongues, mimicking the biological tongues, consist of an array of biosensors that recognize and convert particular chemical compositions or biological reactions to electrical signals. While, e-skin is a mini electronic device prepared from a very thin electrical material in combination with elastic and pliable substrates, mimicking the potentials of human skin. E-tongues and e-skin are cutting-edge technologies of B&B devices which have attracted huge attention in the detection of internal life sign processes in living beings. The sensing performance and efficiency of the e-tongues and e-skin devices have been continuously improving for many years through the development and discovery of novel materials and advanced technologies. Numerous CPs have been employed for the preparation of e-tongues and e-skin devices owing to their fascinating and controllable electrical properties, wide-ranging chemical structures, facile and cost-effective preparation method, high environmental stability, and low toxicity. Polypyrrole (PPy), polyaniline (PANI), polythiophene (PT), and poly(3,4-ethylenedioxythiophene) (PEDOT) are common CPs used for fabricating e-tongues and e-skin biosensors.

## 2. Overview of Contributions

This Special Issue collects 10 contributions, including 3 research articles and 7 review papers, providing a valuable resource for readers, scientists, and engineers working at the intersection of polymer science, biosensing technologies, and bioelectronics, ultimately contributing to the advancement of next-generation B&B devices.

First, Cheng et al. proposed a self-adaptive polymer Fabry–Pérot interferometer (PFPI) sensor for ultrasensitive thermal sensing with a wide linear range, achieving a temperature sensitivity of 0.95 nm/°C, which represents an improvement of two orders of magnitude compared to conventional fiber Bragg gratings (Contribution 1). The authors demonstrated ultrahigh-resolution (0.025 °C) scanning thermal field imaging and sensitive human physiological monitoring, including precise body temperature and respiratory rate detection to address the challenge of spectral shifts exceeding the free spectral range due to introducing a local cross-correlation algorithm for accurate wavelength tracking. They also highlighted the dual capability of the PFPI sensor for both microscopic thermal mapping and non-invasive healthcare applications. In another study, Miller et al. used polydiacetylenes (PDAs), a conjugated polymer, to develop a fluorogenic biosensor with tunable sensitivity through tuning and optimizing PDA vesicles structures (Contribution 2). Particularly, they investigated influences of DA hydrocarbon tail and headgroup on the self-assembled vesicle size and stability, polymerization kinetics, and the fluorogenic, blue to red phase transition. The results indicated that longer DA acyl tails generally induced smaller and more stable vesicles, while both the DA acyl tail length and structure of the headgroup can affect the polymerization kinetics and blue-red transition. Vesicles which are formed through decreasing the acyl tail length are more sensitive to energetic stimuli. Headgroup modifications had different effects depending on the structure of the headgroup. Ethanolamine headgroups resulted in vesicles with potentially increased stimuli responsivity. The lower energy stimulus to induce the chromatic transition was attributed to an increase in headgroup hydrogen bonding and polymer backbone strain. Boronic-acid headgroup functionalization led to vesicles that were generally unstable, only weakly polymerized, and unable to fully transform to the red phase due to strong polar, aromatic headgroup interactions. The promising results of this work demonstrate the design of PDA vesicles in the context of biosensing platforms and include a discussion of the past, present, and future of PDA-based biosensing. Bakhshayesh and Li reported a rapid and cost-effective technique for fabricating concave magnetic-responsive hydrogel discs (CMDs) by straightforward pipetting directly onto microscope glass slides (Contribution 3). This approach enables immediate preparation and customization of hydrogel properties such as porosity, magnetic responsiveness, and embedded particles and is adaptable for use with microarray printers. The concave design increased the surface area by 43% compared to conventional hemispherical hydrogels, enhancing diffusion rates and accelerating reactions. By incorporating superparamagnetic particles, the hydrogels become magnetically responsive, allowing for stirring within reagent droplets using magnets to improve mixing. The results of this study demonstrate that CMDs dissolve approximately 2.5 times faster than hemispherical ones, while numerical simulations indicated a 46% improvement in diffusion speed within the hydrogel. The faster biosensor responses can be achieved by particle with lower diffusion coefficients due to the concave design. Moreover, the authors demonstrated that the increased surface area and ease of fabrication of CMDs enable efficient adaptation for various biological and biomedical applications, particularly in point-of-care diagnostics, where rapid and accurate biomarker detection is critical.

Besides interesting researches, several comprehensive reviews have been also published in this issue, including:

Shao and co-authors comprehensively summarized recent advancements in polymer-based gas sensors for the detection of disease biomarkers in exhaled breath (Contribution 4). The gas-sensing mechanism of polymers, along with novel gas-sensitive materials such as conductive polymers, polymer composites, and functionalized polymers was examined in detail. In addition, they also highlighted key applications in diagnosing diseases through detecting specific biomarkers, including asthma, chronic kidney disease, lung cancer, and diabetes. Moreover, current challenges related to sensor selectivity, stability, and interference from environmental humidity, as well as future perspectives on the development of next-generation polymer-based sensors, including the integration of machine learning for data analysis and the design of electronic-nose (e-nose) sensor arrays were discussed, and proposed.

Sun and Gao provided an overview on the progress of conductive polymers, molecularly imprinted polymers, hydrogels, and composite polymers in medical diagnosis, food safety, and environmental monitoring, focusing on their signal amplification mechanisms and structural optimization strategies in electronic transport regulation, molecular recognition enhancement, and antifouling interface design (Contribution 5). Overall, they mainly summarized and discussed detection performance of conductive polymers for developing highly sensitive, stable, and intelligent biosensors. They also indicated future developments of polymer-based amplification systems in multi-parameter integrated detection, long-term wearable monitoring, and in situ analysis of complex samples, providing new approaches to precision medicine and sustainable environmental health monitoring.

Xu et al. provided discussions on advances and innovations in smart nucleic acid hydrogels (SNAH)-based biosensors, and proposed their development strategies and key trends in customization (Contribution 6). Specifically, they systematically summarized molecular recognition modules involved in the construction of SNAHs and subsequently elaborated on the responses of these modules to external stimuli. Moreover, they outlined the research progress of SNAHs in environmental monitoring, food safety, and medical diagnostics. Finally, they provided an integrated perspective on future opportunities and challenges, highlighting the innovative framework for designing SNAH-based biosensors and offering a practical roadmap for next-generation intelligent sensing applications.

Barman et al. presented a comprehensive analysis of the principles, devices, and emerging materials that have shaped the current landscape of single-molecule detection (SMD) (Contribution 7). They discussed a wide range of sensing mechanisms, including surface plasmon resonance, mechanochemical transduction, transistor-based sensing, optical microfiber platforms, fluorescence-based techniques, Raman scattering, and recognition tunneling. This review highlighted the transformative role of advanced nanomaterials as new transduction pathways that enhance signal strength, specificity, and integration into compact and wearable biosensing platforms. The multifaceted applications of SMD across biomedical diagnostics, environmental monitoring, food safety, neuroscience, materials science, and quantum technologies were also emphasized. The authors proposed future research directions involving multimodal transduction, AI-assisted signal analytics, surface passivation techniques, and modular system design for field-deployable diagnostics. This review offered a comprehensive roadmap for the next generation of SMD technologies, poised to impact both fundamental research and translational healthcare

Phung and Tran presented a critical review of the key characteristics of fluorescent sensors and highlights several common types of conjugated polymers (CPs) used in their design and fabrication (Contribution 8). They summarized and discussed the main sensing mechanisms, state-of-the-art applications, and recent innovations of CP-based fluorescent sensors for detecting target compounds in environmental and biological fields. Moreover, the authors emphasized potential strategies and future perspectives for designing and developing high-performance CP-based fluorescent sensors. This review provided consolidating current scientific evidence to support the advancement of highly sensitive fluorescent sensors based on various CP nanoparticles for environmental and biological applications.

Kamal et al. provided an overview of intrinsic properties of carbon quantum dots (CQDs) such as their excitation-dependent emission, biocompatibility, and quenching properties (Contribution 9). Diverse strategies for easy synthesis are divided into bottom-up and top-down approaches and detailed herein. In particular, the authors highlighted luminescence properties of CQDs, including quenching mechanisms that could even be utilized for the precise and rapid detection of biomolecules. They also discussed methodologies for the mitigation of fluorescence quenching, which is pivotal for the application of CQDs in biosensors and bioimaging.

Meng et al. highlighted the previously examined metal–organic frameworks (MOF)-based nanostructures as well as promising ones for the electrochemical determination of sweat biomarkers (Contribution 10). They concluded that the presence of NPs strongly improves the electrical conductivity of MOF structures, which are known for their poor conductivity. The use of Cu-MOF and Co-MOF nanostructures for detecting sweat biomarkers with the lowest detection limits was discussed. This review demonstrated that different electrochemical methods, such as amperometric, voltammetric, and photoelectrochemical, were successfully used for monitoring the signal of sweat biomarkers.

## 3. Conclusions and Outlooks

Over the past decades, polymer-based B&B devices have been regarded as one of the most important technologies for rapid and accurate diagnosis of virus biomarkers, especially COVID-19. The development of innovative technologies and the discovery of novel polymer nanostructures for the fabrication of B&B devices have attracted significant attention in community and researchers. Consolidating the existing scientific evidence on the use of polymers for the fabrication of flexible and wearable biosensors plays an important role in expanding their practical applications. Moreover, the challenges and limitations of polymers in biosensors also need to be indicated to provide new ideas for future research. Therefore, we are launching the present Special Issue to gather potential studies, including research articles and reviews, on the application of natural or synthetic polymers, conjugated polymers, copolymers, polymer nanoparticles, and polymer composites in the designs and applications of biosensors and bioelectronics. The works compiled in this Special Issue not only highlight the current state of the art but also are expected to inspire the next generation of biosensor technologies, enabling precise diagnostics to be delivered directly at the patient’s bedside.
